# Comparative genome-wide analysis of *Ovis aries* in Saudi Arabia highlighting inbreeding and genetic isolation of the Najdi sheep breed

**DOI:** 10.3389/fgene.2025.1646127

**Published:** 2025-09-29

**Authors:** Abdulrahman K. Aldawish, Mohanad A. Ibrahim, Faisal M. Alsubaie, Quaiser Saquib, Mohammed Fahad Albeshr

**Affiliations:** ^1^ Zoology Department, College of Science, King Saud University, Riyadh, Saudi Arabia; ^2^ National Livestock and Fisheries Development Program, Riyadh, Saudi Arabia; ^3^ Ministry of Environment, Water, and Agriculture (MEWA), Riyadh, Saudi Arabia; ^4^ Data Science Program, King Abdullah International Medical Research Center, Riyadh, Saudi Arabia

**Keywords:** *Ovis aries*, Najdi, Naemi, Harri, single-nucleotide polymorphism genotyping, genetic diversity, SNP, Saudi Arabia

## Abstract

**Introduction:**

Sheep (*Ovis aries*) farming through traditional practices plays a vital role in the socio-economic development of the Kingdom of Saudi Arabia (KSA). Genetic relationships among KSA sheep breeds remain poorly characterized.

**Methods:**

In this study, we performed a comparative genome-wide single-nucleotide polymorphism (SNP) analysis of three sheep breeds (Najdi, Naemi, and Harri) to evaluate their genetic diversity. Blood samples from 95 individuals were genotyped using OvineSNP50 BeadChip, generating 34,026 high-quality SNPs.

**Result and discussion:**

Minor allele frequency (MAF) distribution revealed the highest genetic diversity in Naemi, followed by Harri and Najdi. The run of homozygosity (ROH)-based inbreeding coefficient (F_ROH_) identified Najdi as the most inbred (F_ROH_ = 0.053), indicating its historical isolation, while Naemi displayed minimal recent inbreeding (F_ROH_ = 0.003). The heterozygosity-based inbreeding coefficient (F_IS_) was highest for Najdi (F_IS_ = 0.092), followed by Harri (F_IS_ = 0.027), indicating a greater level of inbreeding, while Naemi (F_IS_ = −0.055) showed signs of outbreeding.

**Conclusion:**

Principal component (PC) and admixture analyses distinctly separated Najdi from the other two breeds, reflecting its unique genetic identity. Wright’s fixation index (F_ST_) further affirmed genetic differentiation between Najdi and Naemi. Moreover, 336 high-F_ST_ SNPs were identified, which were linked to breed-specific genetic signatures. Our study reveals genetic diversity among local breeds and highlights the need for conservation efforts for the Najdi sheep.

## Introduction

The livestock segment is a key economic component of the Kingdom of Saudi Arabia (KSA), with prominent reliance on domestic production and imports to meet the high demand of sheep meat in daily food habits. Nearly 80% of the rural population living in the remote villages of KSA is actively engaged in animal husbandry, participating as nomadic pastoralists, agro-pastoral communities, or through involvement in the livestock value chain (Aldosari, 2018). The harsh climatic conditions and low soil quality limit the diversification of the farming system in KSA. Hence, farmers have widely adopted to practice the rearing of sheep and goats, which also provides stability for small farm establishments. The recent livestock data released by the General Authority of Statistics indicate that KSA is home to 20.5 million sheep, of which 19 million were categorized under traditional holdings, playing a vital role in the country’s agricultural economy (MEWA, 2023).

KSA has state-of-the-art facilities implemented for the commercial production of poultry and dairy farms. Sheep rearing is predominatly practiced using traditional methods due to the adverse agro-climatic conditions. Despite these challenges, it provides direct employment to many people and helps sustain Saudi Arabia’s sociocultural heritage (MEWA, 2021; FAO, 2023b; Alhafi-Alotaibi et al., 2025). Sheep production through organic projects constantly decreased from 16.07 million in 2017 to 12 million in 2021. This decline has been attributed to limited access to pastures, high feed costs, traditional farming practices, and the impacts of climate change (Alhafi-Alotaibi et al., 2025). Despite ongoing efforts, the acceptance of modern breeding systems by traditional sheep breeders remains a significant challenge in KSA. To fill the production gap, under the umbrella of VISION 2030 of KSA, the Sustainable Rural Agricultural Development Program (Reef) (2019–2025) was launched, aiming for transformation in animal husbandry practices (MEWA, 2021). As a result of these ongoing national initiatives, Saudi Arabia achieved a 61% level of national self-sufficiency in red meat production in 2023, reflecting growing domestic capacity to meet food security targets (MEWA, 2023). In this context, understanding and conserving the genetic and productive traits of local breeds becomes increasingly important. Among the different indigenous sheep breeds, the Najdi is believed to have originated in central KSA. It is now also found in the eastern regions of the country and along the border with Kuwait. The breed is known for its distinctive characteristics, economic value, and historical significance (Alamer and Al-hozab, 2004). Phenotypically, Najdi sheep can be identified by their black bodies with white heads, black and white rings around the neck, and their legs and tails have white spots on their legs and tails. They have long legs, fatty tails, convex noses, and pendulous ears. Their average weight and height are 62 kg and 86 cm for male sheep and 45.5 kg and 76 cm for female sheep. Najdi is well adapted to tolerate water shortages and drought and is highly suited to living under desert conditions. Traditionally, the Najdi was reared for its milk and wool but is now more in demand for its meat (MEWA, 2021; FAO, 2022).

Naemi is another well-known breed of sheep that is fully adapted to the semi-arid or arid conditions of KSA and the surrounding Gulf countries. Naemi sheep are fat-tailed and are distinguished by their unique dark brown heads, ears, and necks. They were chiefly bred for meat; however, they also possess greater potential for milk production and exhibit outstanding drought resistance (Alamer and Al-hozab, 2004; FAO, 2022). The height of rams and ewes of Naemi varies between 60 and 80 cm and 65 and 70 cm, respectively, while body weight ranges between 60 and 90 kg for rams and 30 and 50 kg for ewes. In contrast, Harri is another well-regarded breed of sheep in KSA, primarily raised for meat production, and it is a fat-tailed and coarse-wooled breed named after the volcanic Harat region in the northwest of the country. Being well adapted for high temperatures and poor diet, Harri is most prevalent in regions like the Sarawat Mountains, Tehama, the Hejaz plains, and Qassim and is now also found in Jeddah, Makkah, and Madinah (FAO, 2022). The Harri breed stands out from other regional breeds due to its mostly white body and head, the lack of horns in male sheep, and a high twinning rate. Its average body size is medium; height and weight range from 60 to 70 cm and from 40 to 60 kg, respectively. Among the abovementioned three breeds, Najdi is large-sized, with body measurements approximately 10.9% larger than Naemi and 11.4% larger than Harri sheep, making them the largest among local breeds and reinforcing their economic importance in meat and wool production (El-Zarei et al., 2023).

The recent data released by FAO on the risk status of the world’s mammalian breeds indicated an accelerated decrease in global biodiversity, including 30% of sheep breeds at the largest proportion at risk and 107 breeds of sheep already extinct globally (FAO, 2023a). Indigenous sheep breeds thriving under harsh conditions, tolerate nutritional fluctuations, and resist diseases and parasites are valuable resources—not only for understanding their genetic adaptation and biodiversity but also for supporting their biological conservation (Adam et al., 2015). Despite the prominence of Najdi, Naemi, and Harri in KSA, studies encompassing their genetic diversity and conservation are less studied. Microsatellite analysis of Najdi, Naemi, and Harri breeds exhibited considerable genetic polymorphism among them (Musthafa et al., 2012; Al-Atiyat et al., 2018). RAPD analysis has attributed Najdi as a distinct breed from Naemi and Harri (Adam et al., 2015). A recent study on the molecular profiling between Najdi and Naemi demonstrated significant activation of heat tolerance genes (*HSP90AB1*, *HSPB6*, *HSF1*, *STIP1*, *HSP60*, *HSP90*, and *HSPB1*) in Najdi sheep (Samara et al., 2024). Meanwhile, Naemi showed lower within-breed homogeneity than Harri, indicating higher genetic variability (Sabir et al., 2013). Overall, Najdi, Naemi, and Harri breeds in KSA showed high diversity. Najdi possessed genetically distinct characteristics, and no bottleneck was observed. Naemi exhibited low homogeneity, but it showed high polymorphism. On the other hand, Harri showed high homogeneity and low inbreeding, highlighting the need for comparative genomic analysis to better understand their population structure and evolutionary history (Mahmoud et al., 2020). To date, no genome-wide SNP analyses have been conducted on these local breeds. A comprehensive review of regional sheep genetics summarized microsatellite-based diversity in Awassi sheep, underscoring the need for genome-wide analyses in KSA breeds (Meydan et al., 2024).

Advancements in genomic technology, particularly single-nucleotide polymorphism (SNP) genotyping, have revolutionized the area of livestock genetics, *viz*., population genetics, genetic diversity, inbreeding coefficients, and selection patterns (Kanaka et al., 2023). SNP-based genome-wide association studies (GWASs) have successfully identified genes associated with economically important traits such as growth rate, carcass quality, and reproductive efficiency in various sheep breeds (Abied et al., 2020). SNP genotyping provides high-resolution insights into genetic variation, offering a more precise approach to selective breeding and conservation efforts (Deniskova et al., 2019). In this study, we aim to perform a comparative genomic analysis of local sheep breeds (Najdi, Naemi, and Harri) through genome-wide SNP genotyping to comprehensively analyze their genetic diversity and population structure. We also investigated their genomic characteristics, inbreeding coefficients, and effective population size to provide essential insights into their adaptation and resilience. The novelty of this work relies on the fact that local sheep breeds in Saudi Arabia have not yet been investigated from the perspective of SNP genotyping.

## Materials and methods

### Collection of blood samples

Blood samples were collected from Najdi (n = 33), Naemi (n = 24), and Harri (n = 39) across different regions in Saudi Arabia. Representative images of the three breeds are shown in Supplementary Figure S8. Under the guidance of a trained veterinarian, ∼5 mL blood was drawn aseptically from the jugular vein and collected in the vacutainer tubes. Ethical clearance (KSU-SE-24-58) for this work was approved by the Research Ethics Committee of King Saud University. Samples were transported to the laboratory at 4 °C, processed for initial DNA isolation within 24 h, subsequently processed for genomic DNA isolation, and stored at −20 °C until further processing.

### Extraction of DNA

DNA was extracted using the Maxwell^®^ RSC Blood DNA Kit (Promega, United States; Cat. AS1400) and the Quantus™ Fluorometer (Promega, United States; Model E6150), following the manufacturer’s protocol. DNA concentration was quantified, and a concentration of 50 ng/μL was ensured to be suitable for downstream genotyping.

### SNP genotyping and quality control

**FIGURE 1:**
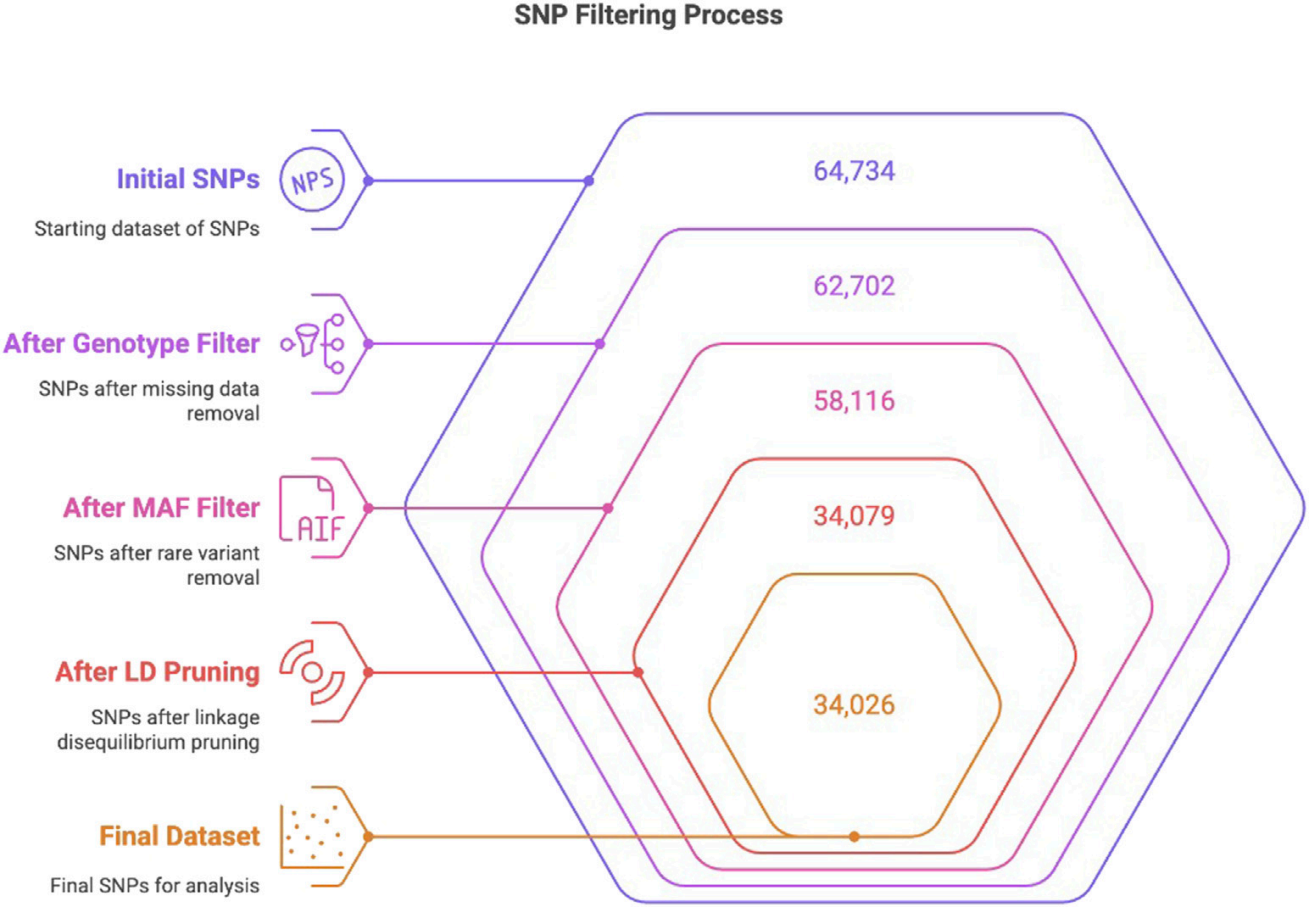
Flow chart depicting the stepwise filtration of the initial 64,734 SNPs to 34,026 through genotype quality filtering, MAF filtering, and LD pruning, following the previously described method (Ajmone-Marsan et al., 2023).

Genotyping was performed using the OvineSNP50 whole-genome genotyping kit (Catalog No. WG-420-1001, Illumina, United States), which captured 64,734 SNP markers distributed across the ovine genome. Genotyping was performed at the Genome Laboratory in the Ministry of Environment, Water, and Agriculture, following the Infinium HTS Assay protocol by Illumina. Data analysis was performed using GenomeStudio software version 2.0.5 (Illumina, United States). Post-implementation of genotype quality filtration, including the exclusion of SNPs with significant missing data, provided 62,702 SNPs. Subsequently, minor allele frequency (MAF) filtration was performed at a threshold of 0.01 to remove rare variants. SNPs that passed QC filters (MAF ≥0.01) generated 58,116 SNPs. Subsequently, linkage disequilibrium (LD) analysis and pruning were performed, resulting in the filtration of 34,079 SNPs. Extreme heterozygosity filtering was performed using PLINK’s (--het) function. One Harri individual (H16) that exhibited extreme observed heterozygosity (Ho) was removed, and the final dataset included 34,026 SNPs across 95 individuals. This final collection of SNPs was preserved for all ensuing analysis (Ajmone-Marsan et al., 2023) (Figure 1).

To compute MAFs for Harri, Naemi, and Najdi breeds, PLINK v1.9 (--freq) was used. Individuals were grouped using breed-specific sample files. MAF values were categorized into four bins, *viz.*, fixed (0.00), rare (>0 to <0.05), intermediate (≥0.05 to <0.10), and common (≥0.10 to ≤0.50). The distribution was visualized using Python 3.10.12 to compare MAF patterns across breeds.

### Run of homozygosity (ROH)

The run of homozygosity (ROH) was identified using PLINK v1.9 (Chang et al., 2015) with the (--homozyg) command, applying customized thresholds to capture both short and long ROH segments. ROH was defined as continuous homozygous regions spanning at least 1 Mb (--homozyg-kb 1000) and containing a minimum of 50 SNPs per segment (--homozyg-snp 50). Restricted gaps between consecutive SNPs to 100 kb (--homozyg-gap 100) were identified to prevent artificial inflation of ROH segments. Additionally, two missing genotype calls per segment (--homozyg-window-missing 2) were allowed to account for possible genotyping errors. The inbreeding coefficient (*F*_*ROH*_) based on the ROH was further determined following the earlier method (Ceballos et al., 2018). To assess the robustness of the results and explore the effect of altering ROH length criteria, the analysis was also utilized using alternate minimum ROH lengths of 300 kb and 500 kb.

### Genetic diversity determination

To assess within-population genetic diversity, observed heterozygosity (*H*_*O*_​) and unbiased expected heterozygosity (*H*_*E*_) were determined using the (--het) command in PLINK (Chang et al., 2015). Additionally, the inbreeding coefficient (*F*_*IS*_) based on the unbiased expected heterozygosity was also estimated (Ajmone-Marsan et al., 2023). The results were analyzed and visualized using Python 3.10.12 to evaluate deviations in heterozygosity across individuals. *F*_*IS*_ was calculated as follows:

$$F_{\mathrm{IS}=}\frac{H_{E}-H_{O}}{H_{E}},$$

where *H*_*E*_ represents the expected heterozygosity and *H*_*O*_ represents the observed heterozygosity.

To estimate the effective population size (*Ne*), LD was calculated using PLINK v1.9 (Purcell et al., 2007; Ajmone-Marsan et al., 2023), based on the *r* ^2^ statistics (Hill and Robertson, 1966). Pairwise LD (*r* ^2^) was calculated within a 1 Mb window using the PLINK flags (--r2 --ld-window-r2 0 --ld-w). The *Ne* trends for each breed were visualized in R 4.3.2 using the ggplot2 package, with initial errors in PLINK flags (--ne) resolved by refining input parameters and modifying the model fitting script.

### Population structure analysis

Principal component analysis (PCA) was performed to assess the genetic relationships among individuals from the three sheep breeds. The genomic relationship matrix (GRM) was constructed, and PCs were extracted to capture population structure patterns. The genomic relationship matrix was calculated following the earlier described approaches (Purcell et al., 2007; Yang et al., 2011). Visualization of PCA results was performed in R 4.3.2 (R-CORE Team, 2021) using statistical and bioinformatics packages, including ggplot2 for graphical representation.

Population structure and individual ancestry proportions were analyzed using sparse nonnegative matrix factorization (sNMF) in the LEA package (Frichot and François, 2015; R-CORE Team, 2021). Genotype data were processed to test K = 1 to 10, with cross-entropy used to identify the optimal K. The best runs were selected based on the lowest cross-entropy values across multiple repetitions. Ancestry proportions (Q-matrices) were extracted for the optimal K-values and visualized using ggplot2. Stacked bar plots illustrate admixture patterns, aiding in the interpretation of genetic relationships and population structure. Additionally, a phylogenetic network was constructed using SplitsTree CE 6.0.0 to visualize relationships among individuals based on genome-wide SNPs. The resulting tree is presented in Supplementary Figure S7.

Wright’s fixation index (*F*_*ST*_) was estimated to quantify the genetic differentiation between populations (Weir and Cockerham, 1984). The calculations were performed using the GRM following the previously described method (Yang et al., 2011). Visualization of *F*_*ST*_ values across populations was conducted in R 4.3.2 (R-CORE Team, 2021) using ggplot2 to generate a heatmap and a Manhattan plot, facilitating the interpretation of genetic differentiation patterns.

### Analysis of shared haplotypes

To assess haplotype sharing, we conducted identity-by-descent (IBD) segment detection using Refined IBD v4.1 (Browning and Browning, 2013; Revelo et al., 2022). Genotypes were phased to infer haplotype structure using Beagle v4.1, and IBD identification was carried out with a 40-SNP window, a minimum segment length of 1.5 cm, and a minimum LOD score of 3. Results were summarized across breed pairs, and Circos visualization was performed using the circlize package in R (Gu et al., 2014).

## Results

### MAF distribution revealed genetic variation among sheep breeds

**FIGURE 2:**
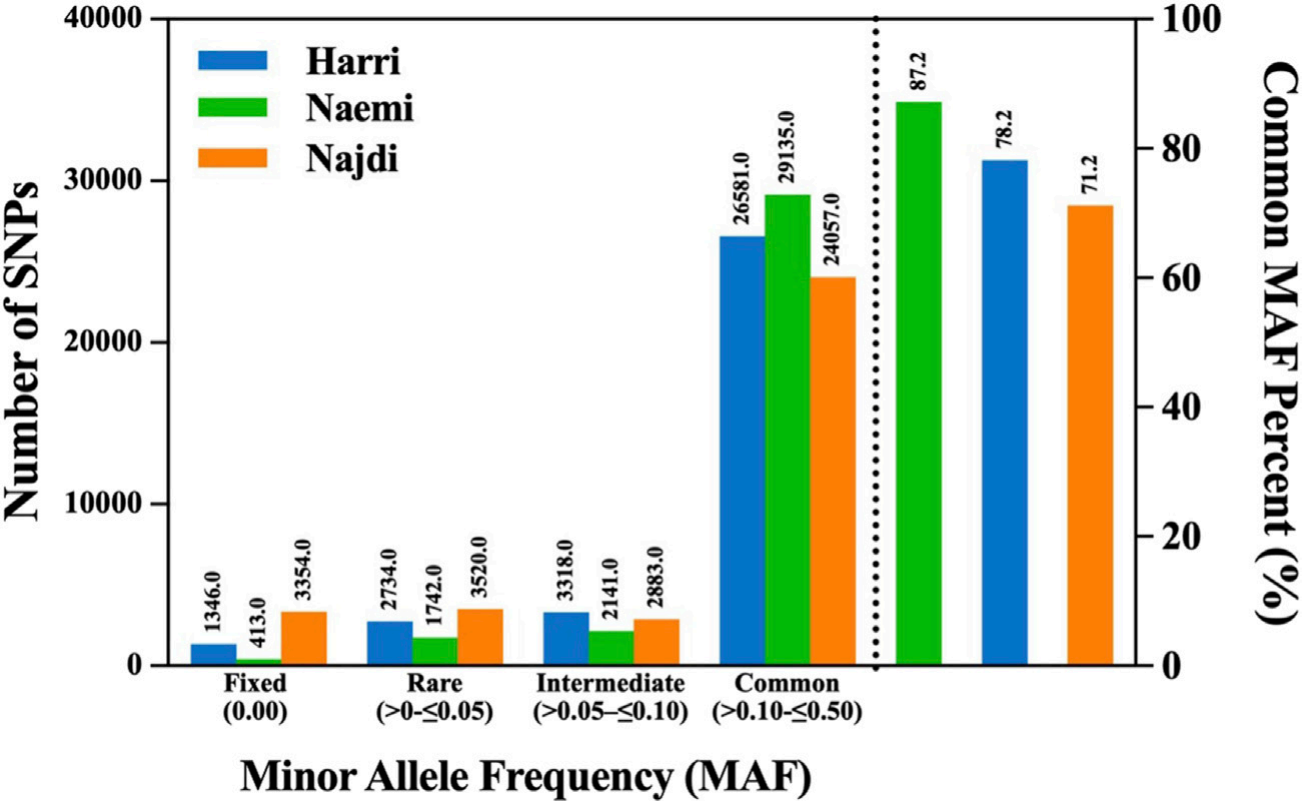
Distribution of SNP counts across different MAF categories Harri (HAR), Naemi (NAE), and Najdi (NAJ).

The MAF distribution exhibited distinct patterns of genetic variations among Harri, Naemi, and Najdi. The majority of SNPs congregated within the common MAF range (≥0.10–≤0.50), with Naemi exhibiting the highest proportion (87.2%), followed by Harri (78.2%) and Najdi (71.2%) (Figure 2; Table 1). Najdi retained the highest proportions of fixed (9.9%) and rare alleles (10.4%). Relatively, Harri showed 3.96% and 10.4% of fixed and rare alleles, respectively. However, the lowest proportions of fixed alleles (1.24%) and rare alleles (5.21%) were found for Naemi. Harri showed the highest intermediate MAF profile (9.76%) among all breeds (Figure 2; Table 1).

**TABLE 1:** Distribution of MAF in three breeds of sheep in KSA.

MAF	Harri (%)	Naemi (%)	Najdi (%)
Fixed (0.00)	3.96	1.24	9.92
Rare (≥0–≤0.05)	8.05	5.21	10.41
Intermediate (≥0.05–≤0.10)	9.76	6.40	8.53
Common (≥0.10–≤0.50)	78.20	87.20	71.20

### ROH exhibited inbreeding patterns in sheep breeds

The total length of ROH and the number of ROH segments were evaluated to determine the genetic diversity and inbreeding patterns in Harri, Najdi, and Naemi (Table 2). The ROH-based inbreeding coefficient (*F*_*ROH*_) was minimal (0.003) in Naemi. Historical inbreeding was more evident in Najdi (0.053) and Harri (0.023) breeds.

**TABLE 2:** ROH and inbreeding coefficient determined in three breeds of sheep in KSA.

Breed	n	Total ROH length (Mb)	Mean ROH count	*F*_*ROH*_
Harri	38	61.78	11.23	0.023
Najdi	33	143.37	21.18	0.053
Naemi	24	7.99	1.96	0.003

Number of individuals (n) and inbreeding coefficient (*F*_*ROH*_) are based on 1 Mb threshold of ROH.

Najdi exhibited the longest total ROH length (143.37 Mb) and the highest mean ROH count per individual (21.18). Harri displayed a moderate ROH profile with a total ROH length of 61.78 Mb. Naemi showed the lowest ROH accumulation (7.99 Mb). Moreover, the data from ROH analysis at lower thresholds of 300 and 500 kb are presented in Supplementary Table S1.

### Heterozygosity analysis indicated the genetic diversity in sheep breeds

**FIGURE 3:**
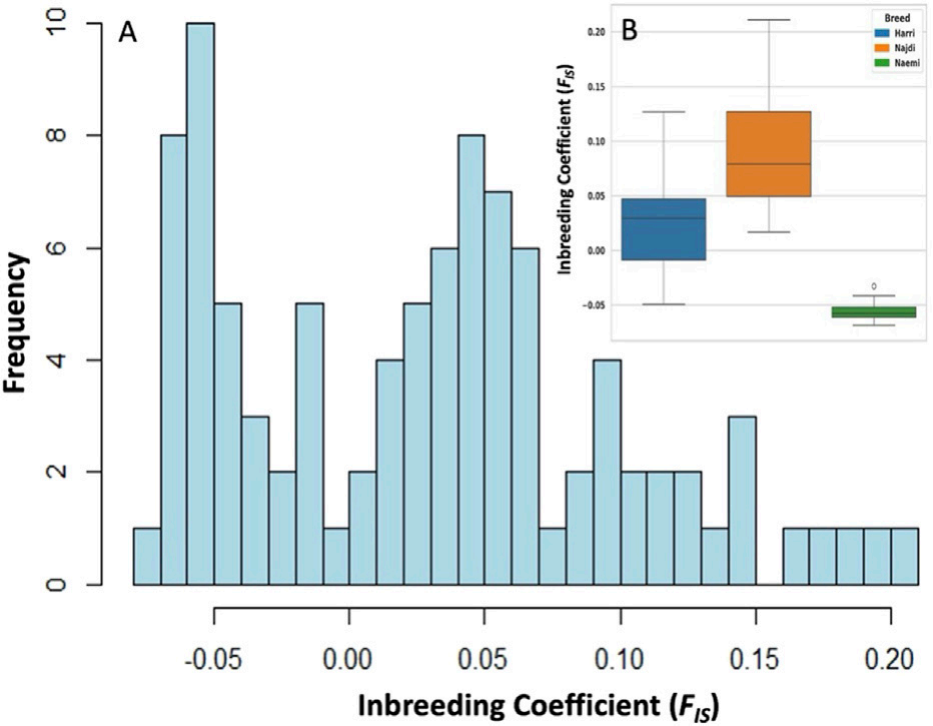
Distribution of inbreeding coefficient (*F*_*IS*_) based on heterozygosity. **(A)** Histograms exhibiting *F*_*IS*_ among all individuals, illustrating variation in genetic diversity of sheep breeds. **(B)** Boxplot depicting *F*_*IS*_ variations among Harri (HAR), Najdi (NAJ), and Naemi (NEA).

The distribution of heterogeneity-based inbreeding coefficient (*F*_*IS*_) across the population followed a bimodal pattern and high-frequency peaks spanning from −0.05 to 0.05. The majority of *F*_*IS*_ values range from −0.05 to 0.20, with a skew toward positive values (Figure 3A). Najdi exhibited the highest inbreeding coefficient (*F*_*IS*_ = 0.092), followed by Harri (*F*_*IS*_ = 0.027) and Naemi (*F*_*IS*_ = -0.055) (Figure 3B). Genetic diversity among Harri, Najdi, and Naemi based on expected heterozygosity (*He*), observed heterozygosity (*Ho*), and effective population size (*Ne*) is shown in Table 3. The lowest effective population size was found in Najdi (*Ne* = 3.30), with a subsequent increase observed in Harri (*Ne* = 6.08) and Naemi (*Ne* = 6.16).

**TABLE 3:** Genetic diversity analysis among sheep breeds.

Breed	n	*Ho*	*He*	*Ne*
Harri	38	0.342	0.352	6.08
Najdi	33	0.319	0.352	3.3
Naemi	24	0.371	0.352	6.16

*Ho*, observed heterozygosity; *He*, expected heterozygosity; *Ne*, effective population size.

### Population structure of Saudi sheep breeds

**FIGURE 4:**
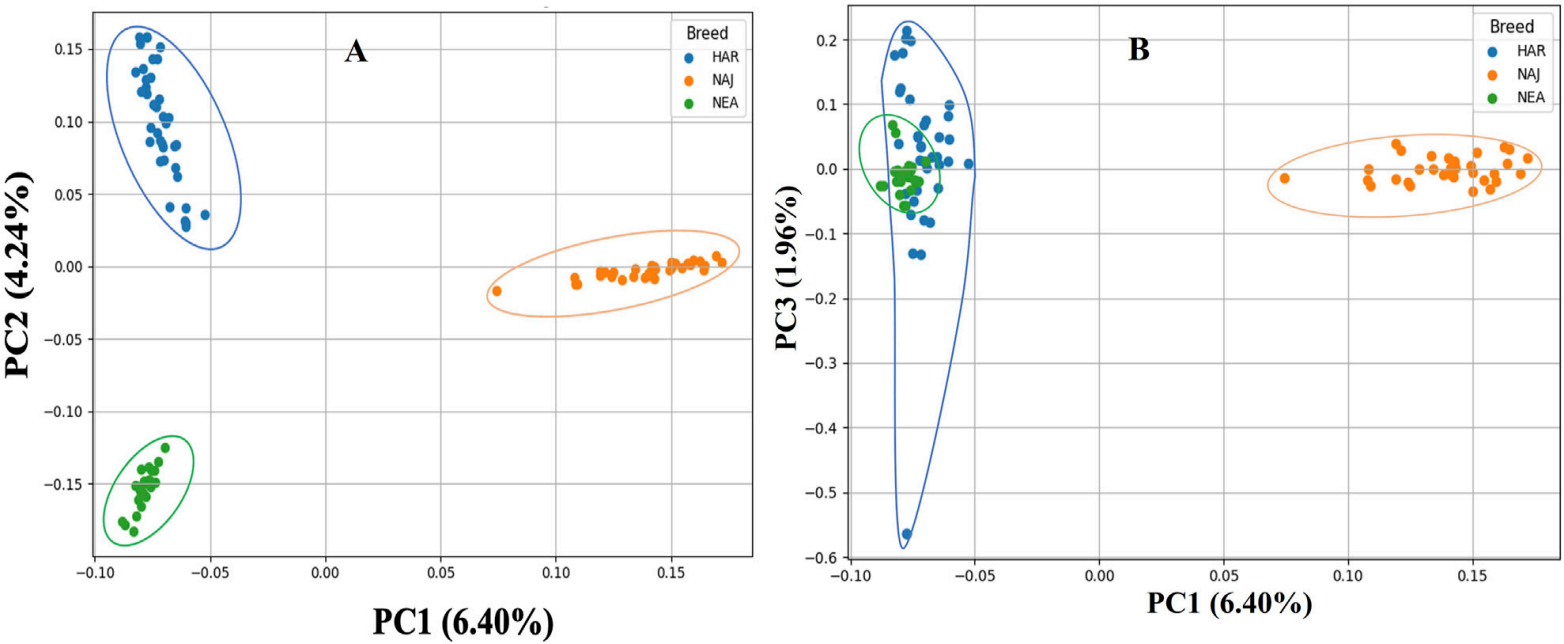
PCA clustering of three sheep breeds of Saudi Arabia. **(A)** PC1 and PC2 analysis reveals genetic differentiation among three distinct populations: Harri (HAR), Najdi (NAJ), and Naemi (NEA). **(B)** PC1 and PC3 exhibited the overlapping of HAR and NEA, while NAJ appeared relatively well separated.

PCA was performed on genome-wide SNP data from 95 individuals representing three distinct breeds of sheep populations. The first 10 principal components explained decreasing proportions of genetic variance, with PC1 (6.40%), PC2 (4.24%), and PC3 (1.96%) collectively accounting for 12.6% of total variation. All components relatively exhibited a small part of the total variance (Supplementary Figure S1,S2). The scatter plot of PC1 vs. PC2 revealed clear separation among the sheep breeds (Figure 4A). Subsequent analysis of the breeds using PC1 vs. PC3 revealed the overlapping of Harri and Naemi, while Najdi appeared relatively well separated. PC1 vs. PC4, PC1 vs. PC5, and PC2 vs. PC3 analyses further showed overlapping patterns between Harri and Naemi (Supplementary Figures S3–S5).

**FIGURE 5:**
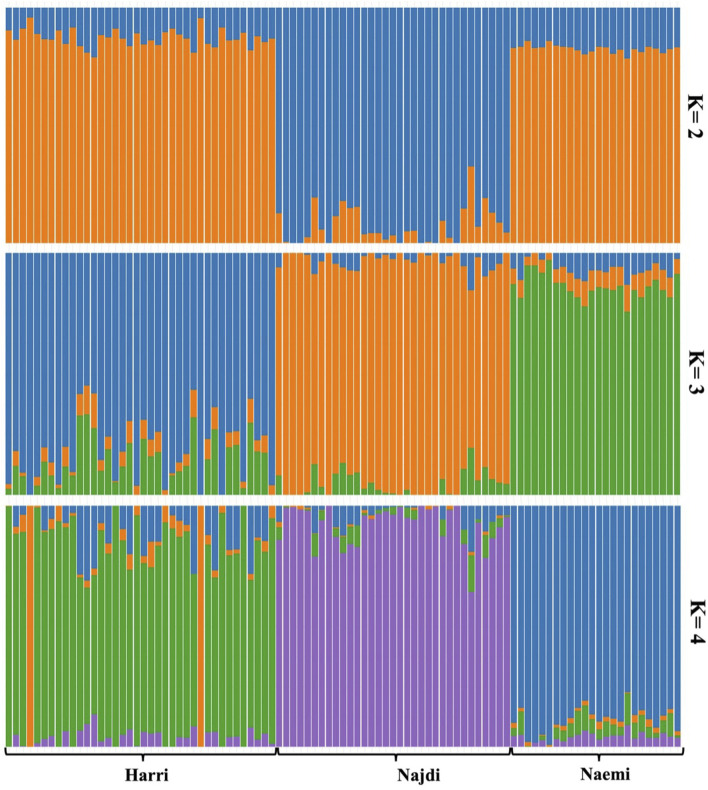
Ancestral population admixture of three sheep breeds in Saudi Arabia analyzed using the LEA package of R.

Admixture analysis was performed using the LEA package at K = 2 to K = 4 levels, which exhibited that K = 4 had the lowest cross-validation error (Supplementary Figure S6). At K = 2, a clear genetic separation of Najdi was observed relative to Harri and Naemi. At this level, Najdi was predominantly assigned to a single cluster (92.79%), while Harri (87.21%) and Naemi (82.58%) were grouped in different clusters (Figure 5). At K = 3, Harri individuals clustered mainly in one group (81.5%), while Naemi sheep were strongly associated with another group (86.14%). The Najdi breed remained genetically uniform (92%). However, at K = 4, each of the three breeds showed a distinct ancestry pattern. In particular, Najdi individuals were assigned to a single unique cluster (92%). Naemi was dominated by a separate cluster (89.03%), while Harri was associated with a distinct cluster (77.31%) but displayed traces of admixture.

**FIGURE 6:**
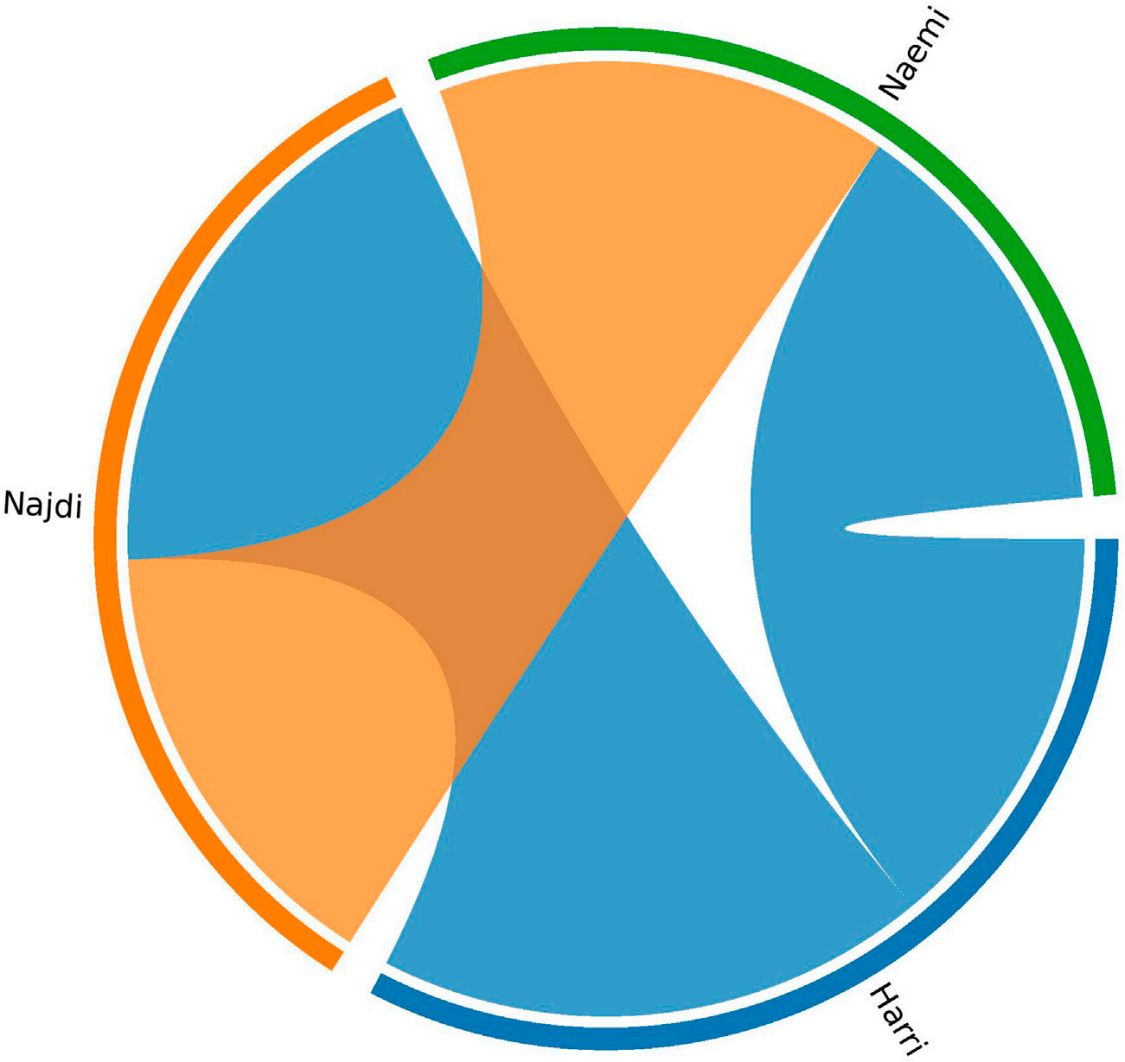
Circos plot showing haplotype sharing among three Saudi sheep breeds based on IBD analysis.

Haplotype sharing analysis revealed varying degrees of IBD segment exchange between the three sheep breeds (Figure 6). The total shared IBD lengths were the highest between Harri and Najdi (673.1 cm), followed by Najdi and Naemi (552.6 cm), and lowest between Harri and Naemi (503.2 cm).

### *F*_*ST*_ analysis affirmed genetic differentiation between breeds

**FIGURE 7:**
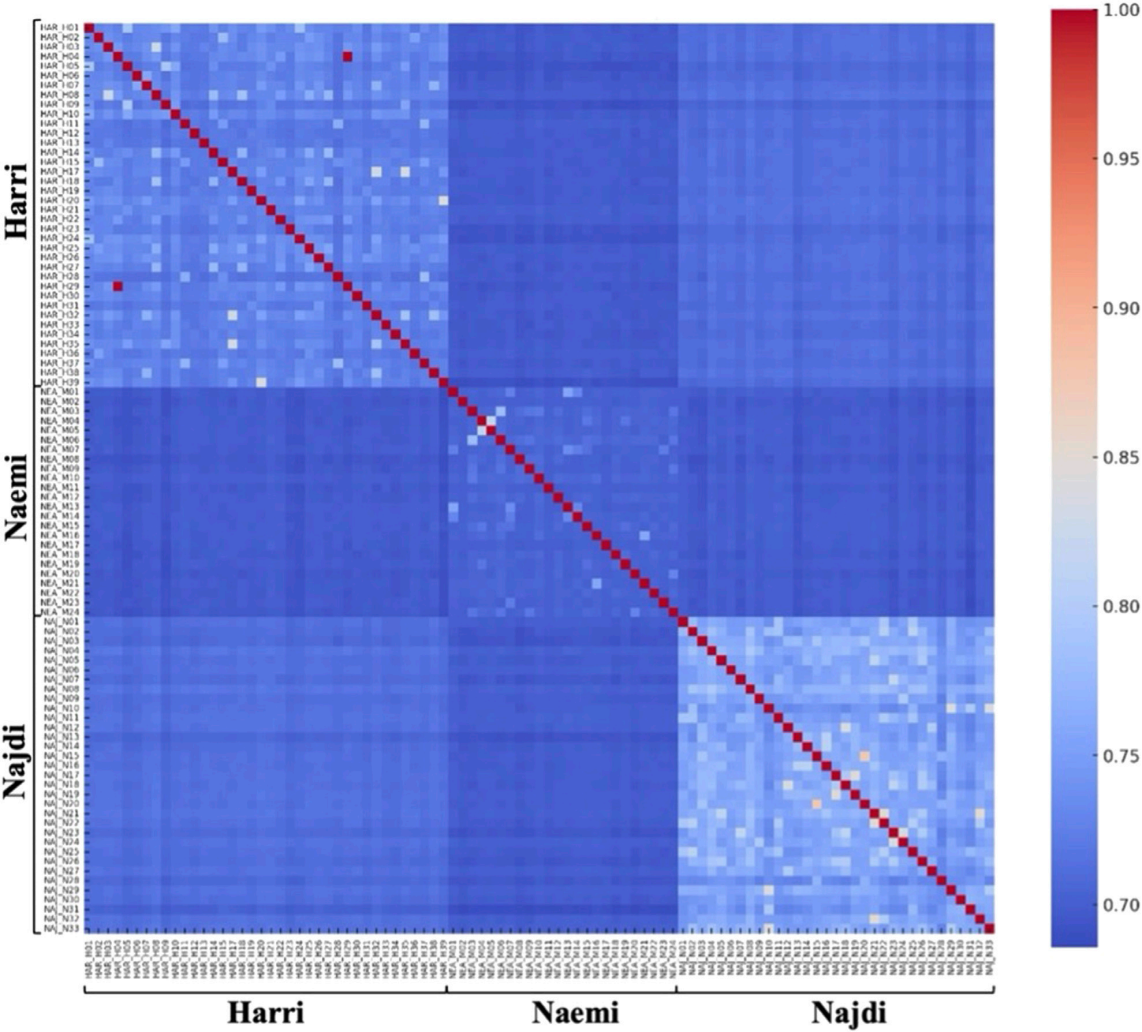
Heatmap showing *F*_*ST*_ pairwise identity-by-state relatedness. *F*_*ST*_ values with higher genetic similarity are indicated in red, and lower similarity in blue.

To explore the genetic relationships among individual sheep and between breeds, a relatedness heatmap was generated using pairwise genomic similarity values for 95 individuals. Distinct block-wise clustering was observed, and a gradient of blue and red regions based on *F*_*ST*_ values reflects lower and higher similarity across breeds (Figure 7). Moreover, the pairwise *F*_*ST*_ values reveal moderate levels of genetic differentiation among the three breeds. The highest *F*_*ST*_ value was found between Najdi and Naemi (0.1071), followed by Najdi and Harri (0.0999). The lowest *F*_*ST*_ value was observed between Harri and Naemi (0.0671) (Supplementary Table S2).

**FIGURE 8:**
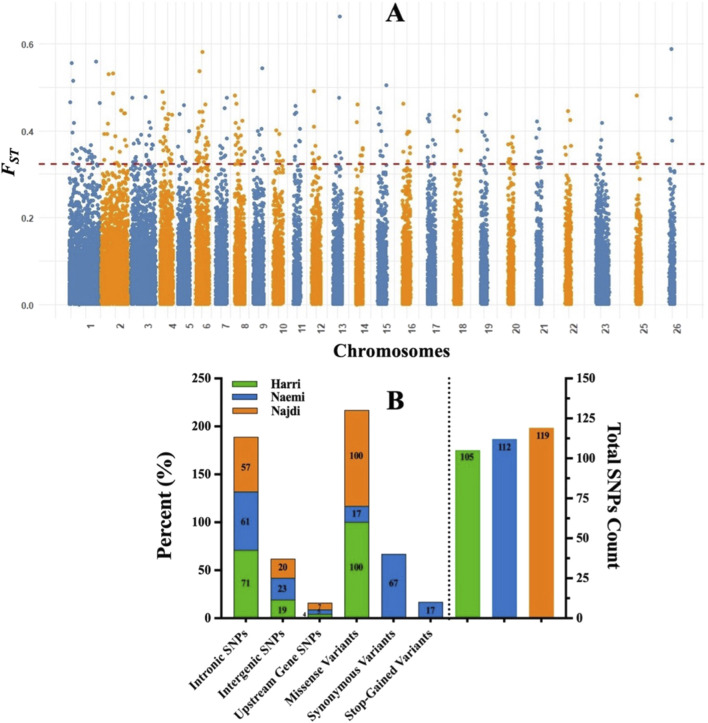
**(A)** Manhattan plot of *F*_*ST*_ values across chromosomes. Each dot represents an SNP with an *F*_*ST*_ value on the Y-axis and genomic position on the X-axis. The red line marks the threshold (*F*_*ST*_ = 0.99). **(B)** Distribution of SNP consequences across Harri, Naemi, and Najdi breeds.

To further investigate the genomic regions contributing to *F*_*ST*_ differentiation, the Manhattan plot was developed (Figure 8A). The plot displays *F*_*ST*_ values across autosomes, with several SNPs exceeding the threshold (*F*_*ST*_ = 0.99), suggesting potential loci under breed-specific selection. A total of 336 high *F*_*ST*_ SNPs were identified. In particular, 119 SNPs for Najdi, 112 SNPs for Naemi, and 105 SNPs for Harri were found. Intronic SNPs were predominant in all breeds, with the highest proportion in Harri (71%), followed by Naemi (61%) and Najdi (57%). Intergenic SNPs accounted for approximately 19%–23% of the total SNPs, with Naemi showing the highest proportion (23%). Upstream gene SNPs were relatively low across all breeds, with Najdi having the highest percentage (7%) compared to Naemi (5%) and Harri (4%) (Figure 8B).

## Discussion

We have performed genomic analysis of three local sheep breeds (Najdi, Naemi, and Harri) of KSA, aiming to evaluate differences in their genetic diversity, population structure, inbreeding coefficients, and effective population sizes to gain key insights into their adaptive potential and genetic resilience. Relative to previous findings using microsatellites, our approach using the genome-wide SNP genotyping has offered novel insights, unraveling a holistic view of the genetic diversity of above the breeds (Al-Atiyat et al., 2018; Mahmoud et al., 2020; El-Zarei et al., 2023).

The distribution of MAF indicated a unique pattern of genetic variations across the three sheep breeds. In particular, Naemi exhibited the highest frequency of common SNPs, which accounted for 87.2%. On the other hand, Harri and Najdi showed 78.2% and 71.2%, specifying a balanced allele distribution and enhanced genetic diversity in Naemi. Although Najdi exhibited greater frequencies of rare and fixed (10.41% and 9.92%) allele distribution, Harri showed 8.1% and 3.96% frequencies, respectively, indicating decreased heterozygosity and putative bottlenecks in Najdi. In addition, Harri showed intermediate patterns of MAF SNPs pointing toward a moderate level of genetic diversity. Nonetheless, our study exhibited an overall mean of 5.04% fixed alleles across populations, which is lower than the average 8.10% of fixed alleles in five sheep breeds of Ethiopia/Africa (Adilo, Arsi-Bale, Blackhead Somali, Horro, and Menz) (Edea et al., 2017) but higher than Merino (3%) and Corriedale (4%) sheep breeds (Grasso et al., 2014). Hence, the accumulation of fixed alleles typically indicates reduced gene flow and historical isolation. The low fixation level in Naemi indicates potential genetic introgression or outbreeding.

Given the robustness of genomic data over classical pedigree analysis, the values of *Ho* and *He* were determined, aiding in the accurate quantification of inbreeding without the need for pedigree data (Curik et al., 2014; Druet and Gautier, 2017). In this connection, we evaluated the inbreeding coefficient (*F*_*IS*_) that has exhibited a negative value (−0.055) for Naemi, while Harri and Najdi showed *F*_*IS*_ of 0.027 and 0.092, respectively. The observed data for Harri and Najdi were *F*_*IS*_ > 0, which clearly indicated that both of them have inbreeding in their population, as also evident by lower values of *Ho* (0.342 and 0.319). In contrast, Naemi has *F*_*IS*_ < 0 [*Ho* = 0.371 higher than *He* (0.352)], affirming that its population has greater heterozygosity than the expected population, such as with outbreeding or cross-breeding (Ajmone-Marsan et al., 2023).

The limitations of *F*_*IS*_ to determine inbreeding in more ancient populations have further prompted us to investigate the genomic coverage using the ROH, which was selectively used to analyze inbreeding when SNPs and WGS data are available. Using frequency and length of ROH, understanding population history, including bottlenecks, and identification of selection signatures were advantageous (Bruford et al., 2015; Ceballos et al., 2018; Meyermans et al., 2020). The total length of ROH in Najdi and Harri was quite long, which clearly indicated recent inbreeding and low genetic diversity in these breeds, possibly due to deleterious homozygous variants (Pryce et al., 2014; Peripolli et al., 2017). In contrast, the total length of ROH in Naemi was low, signifying a historical reduction in their population size, with little recent inbreeding and greater genetic diversity (Peripolli et al., 2017). Najdi, Naemi, and Harri ROHs revealed specific variations in their inbreeding pattern. Among the three breeds, Najdi and Harri showed the highest inbreeding coefficients of *F*_*ROH*_ = 0.053 and 0.023, respectively, indicating historical isolation or intensive selection. Similar to these patterns, high *F*_*ROH*_ values (0.06 and 0.14) were also reported for Czech sheep breeds (Sumava and Wallachian) (Machová et al., 2023). Moreover, the *F*_*ROH*_ values of Najdi and Harri reflected a comparable level of inbreeding as also found in Sopravissana (0.052), Ovino delle Langhe (0.052), and Valle del Belice (0.067) indigenous sheep breeds of Italy (Persichilli et al., 2021). To characterize the divergence, PCA-based population structure analysis was performed. PC1 vs. PC2 resulted in the unique clustering of all three breeds. PC1 vs. PC3 showed genetic isolation of Najdi, while Harri and Naemi overlapped with each other, indicating a shared genetic background. To verify the ancestral, pure, and hybrid populations, we further performed the admixture analysis (Álvarez et al., 2004; Al-Atiyat et al., 2018). It is clearly evident that nearly all individuals of Najdi were characterized as a separate gene pool, forming a consistent, homogenous cluster, affirming their genetic distinctiveness. In contrast, Harri and Naemi showed varying levels of mixing, which is in close agreement with previous reports based on SNP analysis of several regional sheep populations, where gene flow and isolation have led to complex genetic substructures (Ceccobelli et al., 2023; Machová et al., 2023). This pattern is further supported by IBD analysis, where Najdi showed the highest intra-breed haplotype sharing and minimal overlap with Naemi, confirming its genetic isolation. These trends align with previous findings using IBD to detect breed-specific structure in regional sheep breeds (Revelo et al., 2022). In contrast, admixture analysis using 19 microsatellite loci reported close similarity between Najdi and the gene pool of Naemi (Al-Atiyat et al., 2018).

The genetic differentiation between breeds was further determined using *F*_*ST*_, which also separated Najdi from Naemi by a greater value. Harri and Naemi were found closer to each other with low *F*_*ST*_ values (Supplementary Table S2). The analysis of *F*_*ST*_ using identity-by-state relatedness, as evident in the heatmap, further differentiated individual sheep belonging to different breeds. Subsequently, the distribution of SNPs across genomic regions identified 336 highly distinct SNPs, predominantly consisting of intronic and intergenic variants at different loci, signifying breed-specific adaptations to environmental or production-related stresses. Recently, the thermo-physiological and molecular adaptations in Saudi sheep breeds emphasized the advantage of SNP markers as selection signatures to spot heat tolerance traits (Samara et al., 2024).

Although SNP50 genotyping offers high coverage, whole-genome sequencing could reveal rare structural variants. Future studies should integrate phenotype–genotype correlations and prioritize the development of a custom SNP chip for local breeds to refine breeding strategies.

## Conclusion

This study provides high-resolution genomic insights into Najdi, Naemi, and Harri sheep breeds of Saudi Arabia using 50K SNP genotyping. Assessment of key genomic parameters (MAF, *F*_*ROH*_, *He*, *F*_*IS*_, *F*_*ST*_, PCA, and admixture) demonstrated high inbreeding and genetic isolation in Najdi, while Naemi demonstrated the greatest genetic diversity. These findings underscore the urgent need to conserve Najdi sheep in order to preserve their unique genetic heritage.

Future research should integrate whole-genome sequencing, phenotypic assessments, and functional annotation and develop a custom SNP chip to support sustainable breeding and management of Saudi Arabia’s indigenous sheep breeds.

## Data Availability

The data presented in the study are deposited in the EVA repository, accession number PRJEB97998 available at https://www.ebi.ac.uk/eva/?eva-study=PRJEB97998.
