# Supplementary material for: Comparative genome-wide analysis of Ovis aries in Saudi Arabia highlighting inbreeding and genetic isolation of the Najdi sheep breed
Source: Front Genet. 2025 Sep 29;16:1646127. doi: 10.3389/fgene.2025.1646127 (PMC12515495; doi:10.3389/fgene.2025.1646127)
Supplement: Supplementary file 1 [file DataSheet1.docx]

**Supplementary Tables and Figures**

**Table S1.** Summary of the mean ROH segment count, total ROH length, and mean *F_ROH_* for each breed, Harri (HAR), Naemi (NEA), and Najdi (NAJ), at each threshold.

| **Threshold (kb)** | **Breed** | **Mean ROH Count** | **Total ROH Length (kb)** | **Mean FROH** |
| --- | --- | --- | --- | --- |
| 300 | HAR | 0.45 | 34,012 | 0.00036 |
| 300 | NAJ | 0.55 | 49,246 | 0.00060 |
| 300 | NEA | 0.04 | 810 | 0.00001 |
| 500 | HAR | 0.45 | 34,012 | 0.00036 |
| 500 | NAJ | 0.55 | 49,246 | 0.00060 |
| 1000 | HAR | 11 | 61,776 | 0.023 |
| 1000 | NAJ | 21 | 143,366 | 0.053 |
| 1000 | NEA | 2 | 7,996 | 0.003 |


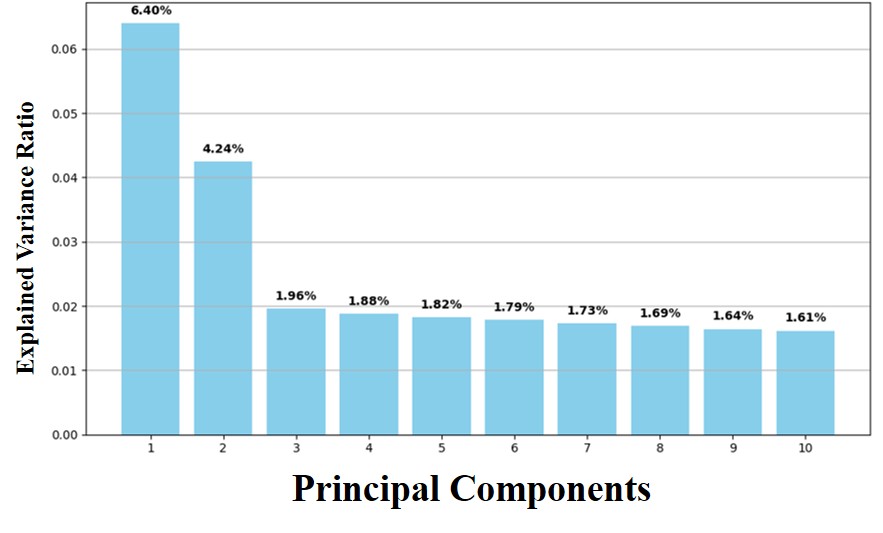


**Supplementary Figure S1:** Bar plot showing the variance explained by the first 10 principal components (PCs) from genome-wide SNP data. PC1 explains the highest proportion (6.40%), followed by PC2 (4.24%), with subsequent PCs contributing progressively less to total variation.


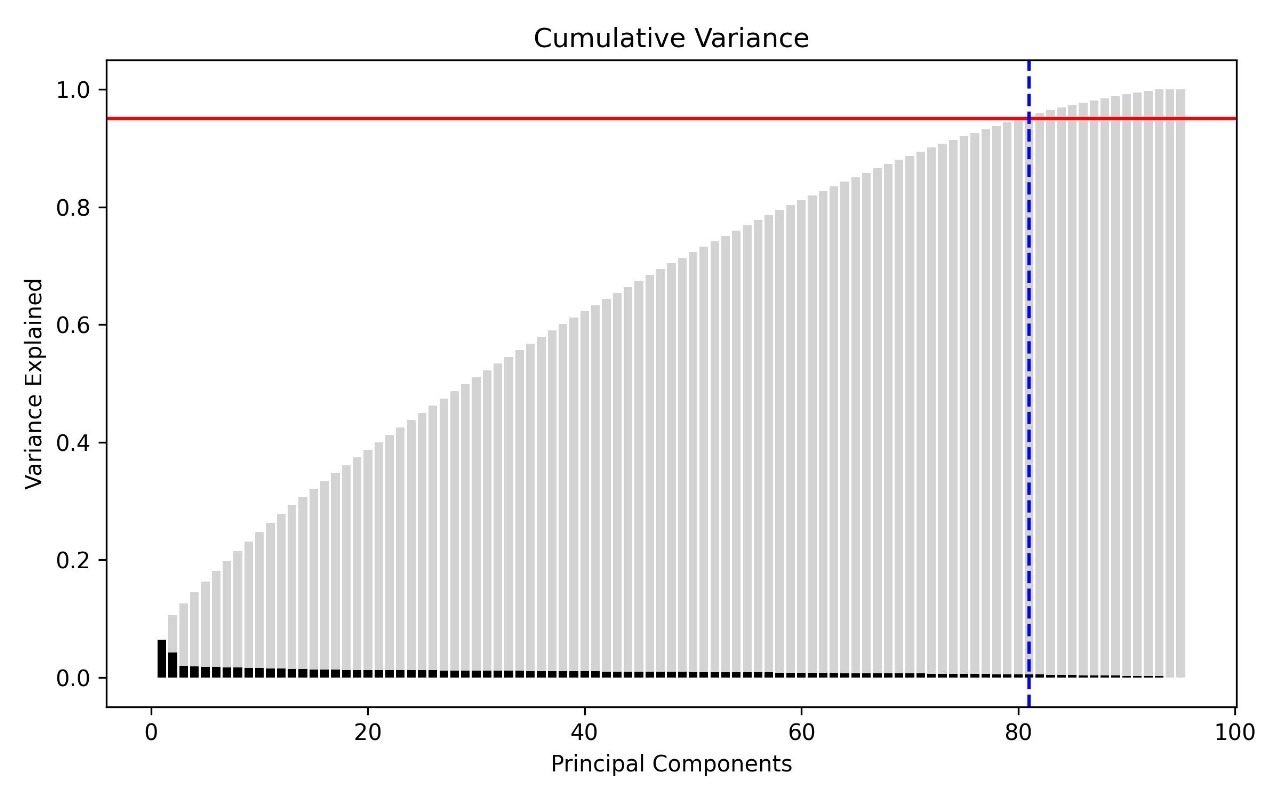


**Supplementary Figure S2:** Cumulative variance explained by principal components (PCs) from genome-wide SNP data. The red line marks 95% variance explained, reached with approximately 81 PCs (blue dashed line).


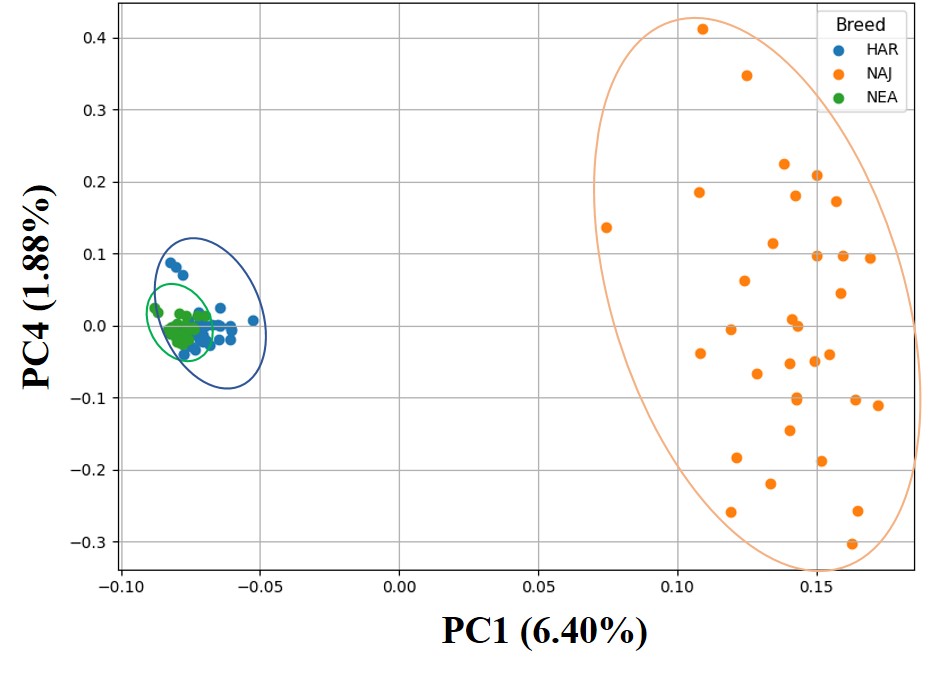


**Supplementary Figure S3.** Principal Component Analysis (PCA) plot of PC1 (6.40%) vs. PC4 (1.88%) showing genetic clustering of three sheep breeds. Harri and Naemi overlap, while Najdi forms a distinct cluster and more within-breed genetic variation.


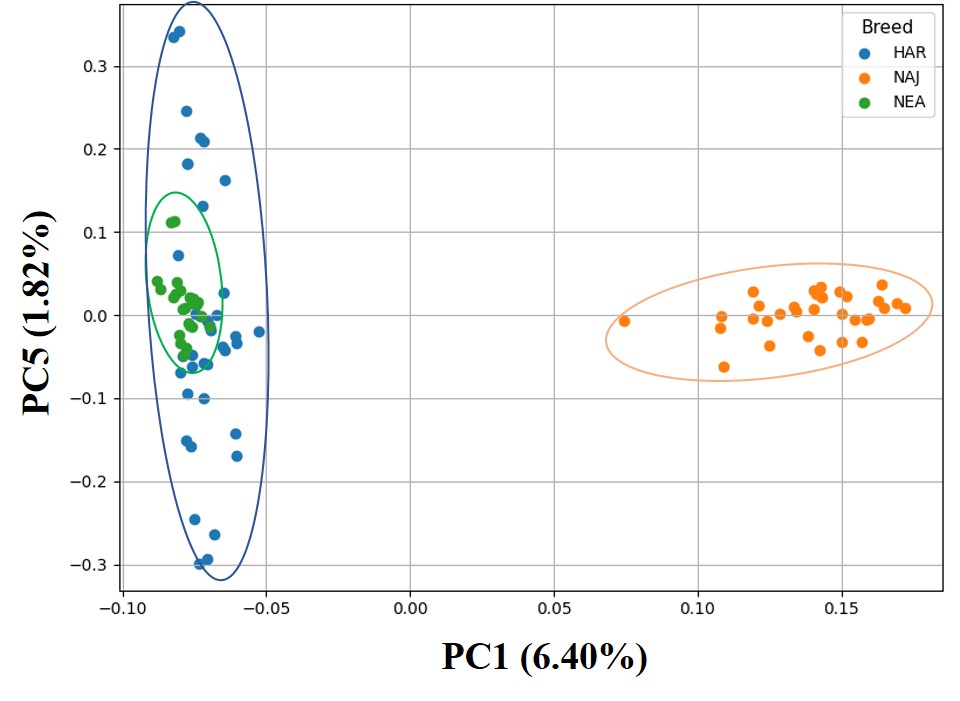


**Supplementary Figure S4.** PC1 (6.40%) vs. PC5 (1.82%) plot displaying genetic structure of three sheep breeds. Harri and Naemi show overlap, while Najdi remains distinctly separated.


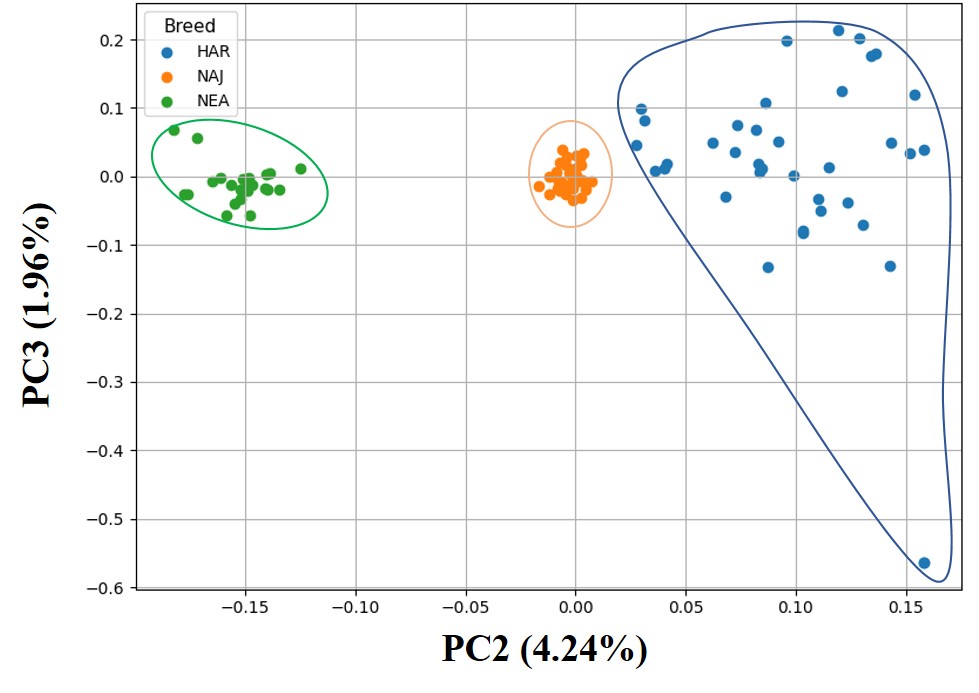


**Supplementary Figure S5.** PC2 (4.24%) vs. PC3 (1.96%) plot showing genetic clustering of sheep breeds. Harri, Najdi, and Naemi form three clearly separated groups. Harri exhibiting more within-breed genetic variation compared to the tightly clustered Najdi.


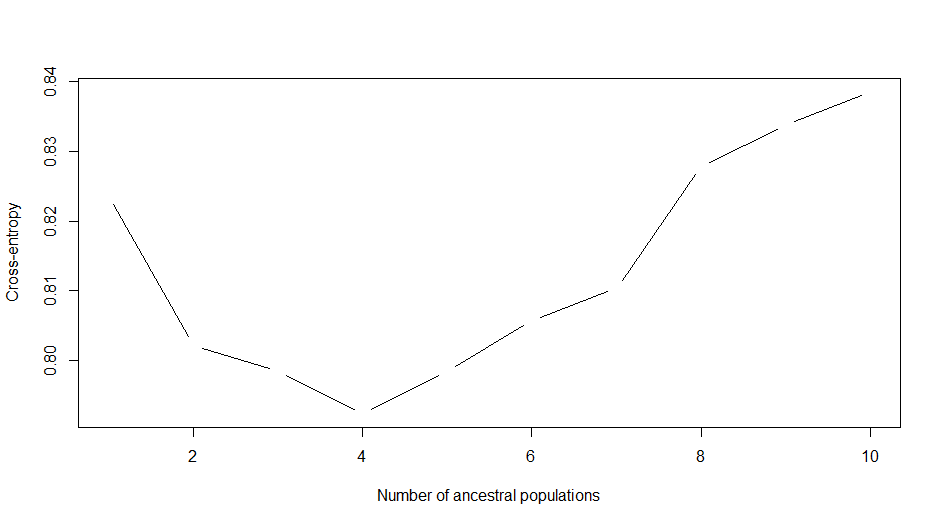


**Supplementary Figure S6.** Cross-entropy plot from an admixture analysis. Cross-entropy is lowest at K = 4, which suggests that 4 ancestral populations best explain the genetic structure

**Supplementary Table S2.** Genetic differentiation (*F_ST_*) between the study sheep breeds based on analysis of 34026 SNPs

|  | **Harri** | **Najdi** | **Neami** |
| --- | --- | --- | --- |
| **Harri** | 0 | 0.0999 | 0.0671 |
| **Najdi** | 0.0999 | 0 | 0.1071 |
| **Neami** | 0.0671 | 0.1071 | 0 |

**Supplementary Table S3.** Pairwise and within-breed haplotype sharing based on identity-by-descent (IBD) segment analysis among Harri (HAR), Najdi (NAJ), and Naemi (NEA) sheep breeds. The table summarizes the number of IBD segments and the total shared IBD length (in centiMorgans, cM) between breed pairs.

| **Breed 1** | **Breed 2** | **IBD Segments** | **Total IBD Length (cM)** |
| --- | --- | --- | --- |
| HAR | HAR | 14,127 | 102,945.36 |
| NAJ | NAJ | 24,677 | 259,606.99 |
| NEA | NEA | 623 | 2,221.69 |
| HAR | NAJ | 154 | 624.78 |
| HAR | NEA | 27 | 64.51 |
| NAJ | NEA | 55 | 161.44 |


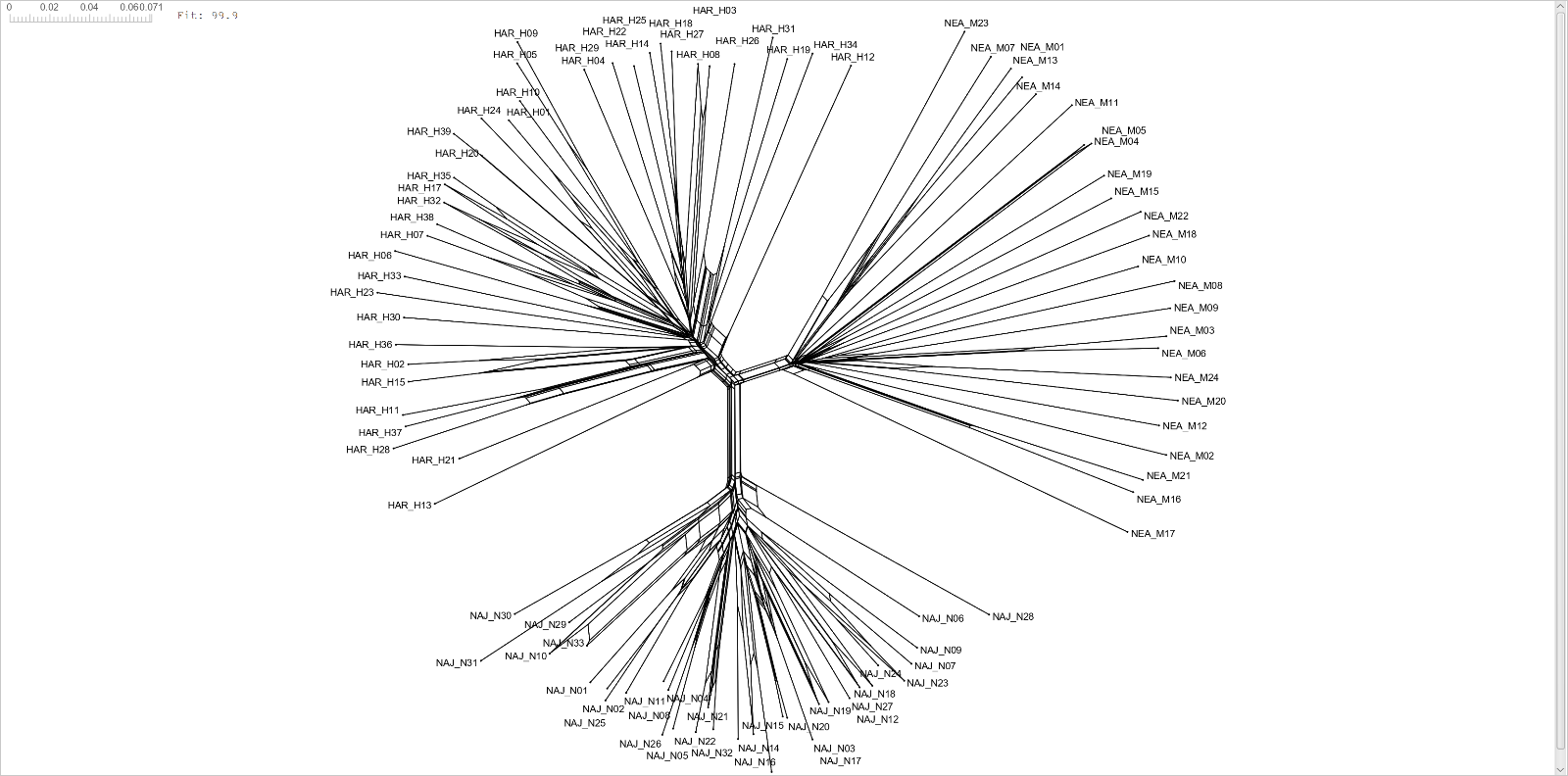


Supplementary Figure S7. Phylogenetic network generated using SplitsTree based on genome-wide SNP data from Harri (HAR), Najdi (NAJ), and Naemi (NEA) sheep breeds. The network illustrates the genetic relationships among individuals, with clear clustering of samples by breed.


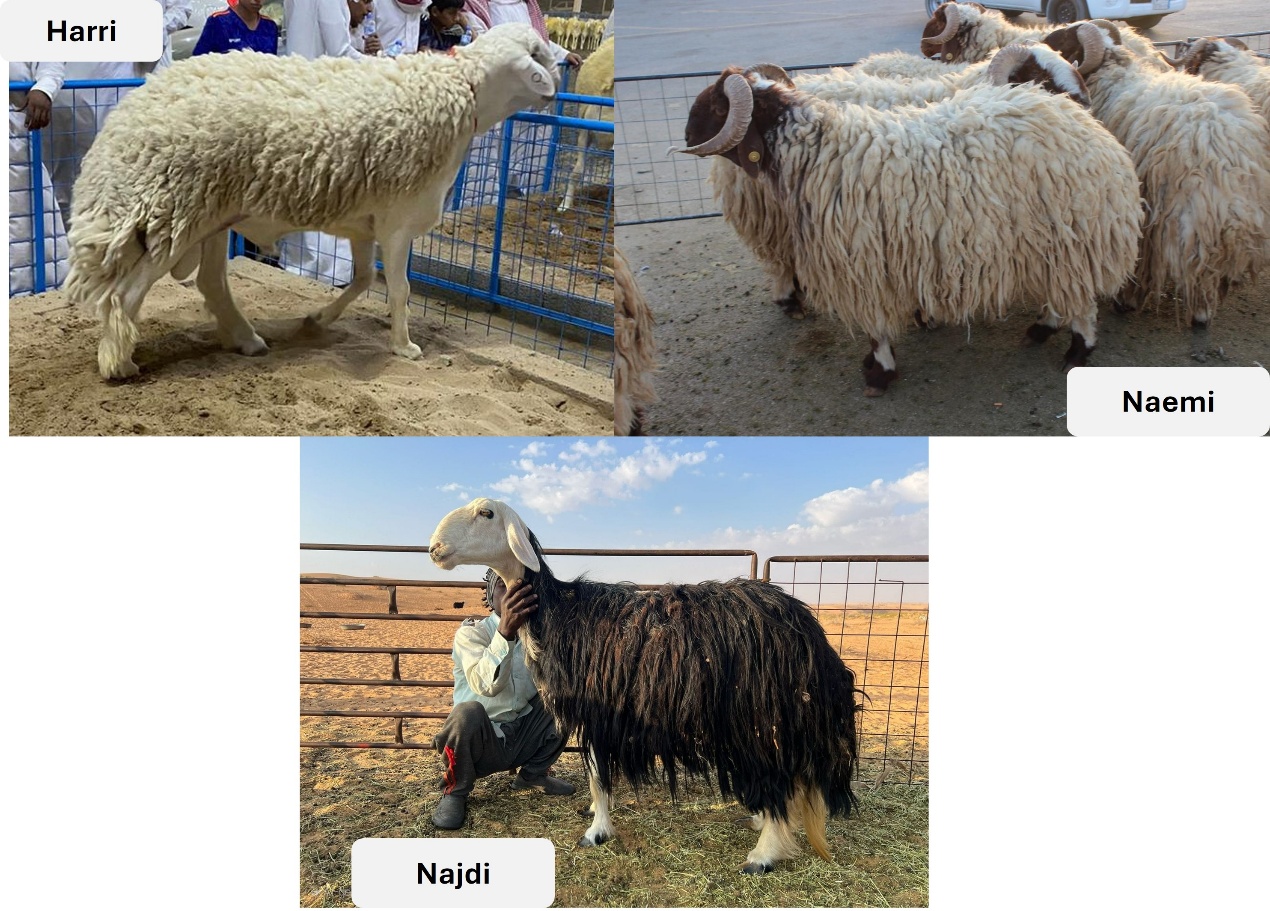


**Supplementary Figure S8.** the three indigenous sheep breeds picture of Saudi Arabia included in this study.
